# Decoding the Effects of Bexarotene treatment on brain of AD-like model mice: Single-Cell Transcriptomics and Chromatin Accessibility Analysis

**DOI:** 10.21203/rs.3.rs-7201032/v1

**Published:** 2025-08-13

**Authors:** Yi Lu, Xuebao Wang, Carolina Saibro-Girardi, Nicholas Francis Fitz, Radosveta Koldamova, Iliya Lefterov

**Affiliations:** University of Pittsburgh; University of Pittsburgh; University of Pittsburgh; University of Pittsburgh; University of Pittsburgh; University of Pittsburgh

**Keywords:** Alzheimer’s Disease, Retinoid X Receptor, Bexarotene, Single-Cell Transcriptomics, Chromatin Accessibility, Transcription Factor Footprinting

## Abstract

**Backgound:**

Ligand-activated Retinoid X Receptors (RXRs) regulate gene networks essential for neural development, neuroinflammation, and metabolism. Understanding how RXR activation influences chromatin architecture and gene expression may reveal therapeutic strategies for neurodegenerative diseases.

**Methods:**

We used Bexarotene-treated APP/PS1ΔE9 mice to study RXR-mediated regulatory mechanisms. To investigate epigenomic and transcriptional effects, we integrated single-nucleus ATAC-seq (snATAC-seq) with single-cell RNA-seq (scRNA-seq) and validated differentially accessible chromatin peaks using RXR ChIP-seq. Transcription factor (TF) footprinting analysis were performed to map regulatory networks activated by ligand-bound RXR.

**Results:**

Our integrated analyses revealed a multilayered transcriptional cascade initiated by a single linear RXR signaling event. We identified RXR-centered regulatory circuits involving heterodimer activation, subsequent upregulation of multiple downstream TFs, and induction of metabolic pathways relevant to neural function. The results of a detailed analysis of TF regulatory networks in neuronal systems suggests that Bexarotene doesn’t dismantle the fundamental regulatory scaffold in neurons but rather modulates RXR regulatory role through existing TF networks.

**Conclusions:**

This study demonstrates that combining scRNA-seq, snATAC-seq, and ChIP-seq enables a comprehensive analysis of RXR-mediated transcriptional regulation. RXR activation orchestrates complex gene networks that may help restore brain homeostasis in the context of amyloid pathology, neuroinflammation, and neuronal injury.

## Background

Nuclear receptors (NRs) are ligand-activated transcription factors (TFs) that play critical regulatory roles in diverse metabolic pathways and cellular processes implicated in the onset and progression of numerous diseases, including neuropathological and neurodegenerative disorders. The human Retinoid X Receptor alpha (RXRα) was first cloned nearly 35 years ago by D. Mangelsdorf using 9-cis retinoic acid (9-cis RA) as its ligand^1^. For decades, 9-cis RA—a stereoisomer of all-trans retinoic acid—was considered the endogenous ligand for RXRα, a view that evolved when R. Rühl et al. (2015) identified 9-cis-13,14-dihydroretinoic acid as a physiologically relevant RXR ligand in mice^2^.

RXRs belong to the NR superfamily and exist as α, β, and γ isoforms, each encoded by distinct genes in both humans and mice and broadly expressed across tissues^3,4^. RXRs function primarily as obligate heterodimer partners for over 20 hormone-responsive NRs, mediating transcriptional regulation through DNA binding and recruitment of coregulatory complexes^5^. These heterodimers are categorized as permissive or non-permissive based on whether RXR ligands alone can activate them. While ligand binding alters RXR’s conformation, non-permissive heterodimers require the partner NR’s ligand for transcriptional activation. The structural principles of RXR heterodimerization and DNA binding were well established by the late 1990s^6^. These studies also elucidated the receptor’s modular architecture, including ligand-binding, dimerization, DNA-recognition, and transcriptional activation domains, as well as its interaction with coactivators and corepressors^7–9^.

A two-step model of RXR heterodimer function was proposed in the early 2000s. The first involves heterodimerization in solution via ligand-binding domains; the second occurs upon DNA binding, where functional activation is contingent on the assembly of coregulator complexes^10^. However, technical limitations at the time prevented genome-wide identification of genes regulated by RXR. It remained unclear to what extent RXR activation beyond known promoter-proximal sites influenced transcription on a broader genomic scale.

The advent of ChIP-seq enabled genome-wide mapping of RXR-binding sites and facilitated the concept of an RXR cistrome, validating the heterodimerization model of RXR-mediated transcription. Despite efforts to construct RXRα interaction networks^5^, these remained incomplete due to the complexity of coregulatory interactions and a lack of integrative tools to analyze genome-wide transcription and chromatin architecture. Only recently, with the development of single-cell multi-omics and powerful bioinformatics pipelines, has it become feasible to build comprehensive TF regulatory networks from such high-dimensional datasets. A recent study integrating over 40 cistromes from multiple labs demonstrated that TF-DNA interactions are often promoter-specific^11^; however, these analyses rarely extended beyond static promoter regions, limiting their capacity to reconstruct dynamic regulatory networks.

Efforts in the late 1990s and early 2000s led to the development of small-molecule ligands targeting permissive RXR heterodimers, notably LXR/RXR^3,4^. Synthetic RXR ligands, or retinoids, are chemically modified vitamin A derivatives designed to enhance therapeutic efficacy and reduce side effects. Widely used in dermatology and oncology, agents like Isotretinoin, Adapalene, and Bexarotene modulate gene expression programs that influence differentiation, proliferation, and apoptosis^12,13^. In dermatology, retinoids treat conditions like severe acne and psoriasis, while in oncology, Bexarotene is approved for treating cutaneous T-cell lymphoma and is under investigation for other malignancies^14^. NRs have also emerged as attractive drug targets in a wide range of liver diseases. Considering intercellular and interorgan cross-talks in development and progression of liver disease, strategies to simultaneously modulate different NRs expressed in distinct liver cell types are considered highly beneficial^15^.

More than two decades ago, RXR activation was proposed as a therapeutic strategy in Alzheimer’s disease (AD)^16^. Given the disrupted cholesterol and phospholipid metabolism in AD, ligand-activated RXR signaling—particularly via LXR/RXR heterodimers—emerged as a promising target. One of the first key genes identified as an RXR-responsive target was ABCA1, a transporter critical for cholesterol efflux. Subsequent studies confirmed ABCA1’s role across multiple neurodegenerative conditions, supporting the inclusion of RXR activation in experimental therapeutics^17–22^. Despite the therapeutic promise, the broader transcriptional consequences of RXR activation in the brain—particularly in the context of chromatin remodeling—remain incompletely understood^23^.

Here, we aimed to characterize the effects of Bexarotene treatment on chromatin accessibility and transcriptional regulation in the brains of AD-like APP/PS1ΔE9 mice. We hypothesized that ligand-activated RXR, acting via heterodimers or homodimers, alters chromatin accessibility to enable the assembly of functional transcriptional machinery at specific genomic loci. To test this, we employed single-cell RNA sequencing (scRNA-seq) and single-nucleus ATAC-seq (snATAC-seq), combined with ATAC-seq footprinting and RXR ChIP-seq, to construct a cell-type-resolved map of transcriptional and epigenomic remodeling. This integrative approach enabled us to reveal how RXR activation orchestrates cell-specific regulatory networks in the AD brain.

## Materials and methods

### Animals and treatment

APP/PS1ΔE9 [B6.Cg-Tg (APPswe,PSEN1dE9)85Dbo/Mmjax] transgenic mice (referred to as APP/PS1) were purchased from The Jackson Laboratory (USA). Heterozygous experimental animals were bred in-house and male and female mice were used for all experiments. All animal procedures were performed in accordance with the guidelines outlined in the Guide for the Care and Use of Laboratory Animals from the United States Department of Health and Human services and were approved by the University of Pittsburgh Institutional Animal Care and Use Committee. All animals were littermates and housed with a 12-hour light/dark cycle with ad *libitum* access to food and water. APP/PS1 mice aged 4 to 6 months were randomly assigned to either treatment group (100 mg/kg/day) or Control group (Vehicle treatment), with paired distribution according to age and sex. Bexarotene (Thermo Scientific Chemicals # J63701MC, CAS 153559-49-0) was prepared in a vehicle solution of corn oil containing 1% DMSO on the day of treatment. The animals received 10 μl per gram of body weight of either bexarotene or vehicle solution by oral gavage for 10 consecutive days.

### Perfusions and brain tissue processing

Mice were anesthetized by I.P. injection of Avertin (1.25% tribromoethanol, 2.5% 2-methyl-2-butanol, 250mg/kg body weight). Blood was collected via cardiac puncture of the right ventricle using EDTA-treated syringes, followed by transcardial perfusion with 20 mL of 0.1M PBS, pH 7.4. The blood was centrifuged to collect plasma for storage. For single-cell assays, one hemisphere was dissected to remove the cerebellum, olfactory bulb, and subcortex. The other hemisphere was drop-fixed in 4% phosphate-buffered paraformaldehyde at 4°C for 48 hours before being transferred to 30% sucrose for storage.

### Tissue dissociation

Freshly dissected mouse brain tissue was immediately dissociated using the Adult Brain Dissociation Kit (Miltenyi, # 130-107-677) following manufacturer’s instructions with minor modifications. Briefly, brain tissue was dissected into small pieces in Hank’s balanced salt solution (HBSS, Gibco, # 14025092), and tissue pellets were incubated in enzyme mix with gentle rotation and mechanical dissociation. The resulting suspensions were filtered and washed. Next, tissue debris were removed using Debris Removal Solution (Miltenyi) following manufacturer’s instruction. Cell pellets were washed once using Dulbecco’s phosphate buffered saline (DPBS) containing 0.04% bovine serum albumin (BSA) and filtered before cell counting. Cell viability was assessed using Trypan Blue, ranging from 85–95%. Approximately 16,500 cells per sample were used for scRNA-seq library generation, with the remainder stored in liquid nitrogen for nuclei isolation and snATAC-seq libraries.

### Library preparation for scRNA-seq

scRNA-seq libraries were prepared using the Chromium Next GEM Single Cell 3’ Kit v3.1 and Chip G Single Cell Kit (10x Genomics) according to manufacturer’s protocol. Cell-RT mix were prepared, targeting at 10,000 cells per sample, and applied to the Chromium Controller (10x Genomics) for GEM generation and barcoding. Samples were then purified from post GEM-RT cleanup and full-length cDNA were amplified for library construction. Libraries were checked by Bioanalyzer High Sensitivity DNA kit (Agilent, USA) and sequenced on Illumina NovaSeq (UPMC Genome Center, Pittsburgh, USA) according to manufacturer’s recommendations.

### Nuclei isolation and library preparation for snATAC-seq

Cryopreserved cells left from scRNA-seq libraries were thawed in 37°C water bath, checked for viability, and incubated with 100 μl lysis buffer (10mM Tris-HCl pH 7.4, 10mM NaCl, 3mM MgCl_2_, 0.1% Tween-20, 0.1% IGEPAL, 0.01% Digitonin, and 1% BSA) for 3 minutes on ice, followed by a washing step (10mM Tris-HCl pH 7.4, 10mM NaCl, 3mM MgCl_2_, 0.1% Tween-20, 1% BSA). Nuclei concentration was assessed using AOPI staining on Countess II (Thermo Fisher Scientific) and adjusted to 1000 nuclei/μl. Droplet-based libraries were immediately prepared after nuclei isolation using the Chromium Next GEM Single Cell ATAC Kit v2 and ChIP H Single Cell Kit (10x Genomics) according to manufacturer’s instructions. Single-nuclei suspensions were incubated with transposase for 30 minutes and used for GEM generation and barcoding on Chromium Controller (10x Genomics) targeting at least 10,000 nuclei per sample. After cleanup, libraries were constructed and indexed using Single Index Kit N Set A (10x Genomics). Bioanalyzer High Sensitivity DNA kit (Agilent) was used for quality control, and pooled libraries were sequenced by UPMC Genome Center (Pittsburgh, USA) on Illumina NovaSeq S2 PE100 according to manufacturer’s recommendations.

### Chromatin Immunoprecipitation and sequencing – ChIP-seq

Chromatin immunoprecipitation and sequencing (ChIP-seq) was performed according to our routine protocol and as before^21,22^. Briefly, brain lysates of mice treated with Bexarotene or Vehicle were sonicated with 3 pulses of 15 sec at amplitude 30, a 120 sec pause and 3 pulses of 15 sec at amplitude 40 using a Model 705 Sonic Dismembrator (Fisher Scientific, Pittsburgh, PA), to obtain fragments of 200–600 bp. For immunoprecipitation, we used a rabbit polyclonal anti-RXR (ΔN 197, #sc-774, Santa Cruz, CA). ChIP libraries were generated using TruSeq ChIP Sample Prep Kit (Illumina, San Diego, CA) following manufacturer’s protocols. For each of the steps, samples were purified by AMPure XP beads (Beckman Coulter, Brea, CA). Adapter-ligated samples were separated on a 2% agarose gel to obtain 250–300 bp size-range of DNA fragment to remove unligated adapters. The libraries were validated by Agilent Technologies 2100 Bioanalyzer to check the size, purity, and concentration of the sample before the sequencing, and sequencing performed on Illumina HiSeq2000 instrument at the Functional Genomics Core, UPenn, Philadelphia (http://fgc.genomics.upenn.edu/).

### Quantification and Statistical analysis

#### scRNA-seq data analysis

Single cell barcoded reads were demultiplexed and aligned to the mouse reference genome (GRCm38) using Cell Ranger pipeline v7.0 (10x Genomics). Cells with over 200 unique molecular identifiers (UMIs) were selected for single cell gene expression analysis. Each of the scRNA-seq libraries were read into R (v4.2.0) and processed using Seurat package^24,25^ (v4.2.1) as previously described^26^. Cells were filtered by the following criteria: (1) unique feature counts > 200 and < 5000, (2) total counts < 50000, and (3) < 10% of mitochondrial gene counts. After filtering, 37,634 cells were kept and SCTransform was used for normalization and scaling. We performed principal component (P.C.) analysis followed by clustering using top 10 P.C.s at 0.25 resolution. Differential expression was performed using “MAST” algorithm^27^ to identify cluster marker genes and help manually annotate cell types for each cluster. Cell types of interest were then subset as individual Seurat objects for further analysis. The mean expression of cell-type specific gene sets was added to the matrices using “AddModuleScore” function, and an additional cell filtering step removed subclusters or cells expressing non-selective gene sets. For differential expression between groups, we used “MAST” algorithm, and a gene was considered differentially expressed if it had a Bonferroni-corrected p value < 0.05.

#### snATAC-seq data processing and cell type annotation

Raw sequencing reads were demultiplexed and mapped onto the mouse reference genome (GRCm38) using “cellranger-atac count” pipeline (10x Genomics). Arrow files were created from fragment files in ArchR^28^ (v1.0.1). High-quality nuclei were selected based on two criteria: (1) Transcription start site (TSS) enrichment score greater than 4, and (2) number of mapped fragments between 1,000 and 100,000. Doublets were identified using the “addDoubletScores” function and filtered with the “filterDoublets” function. Iterative LSI dimensionality reduction and clustering were performed using ArchR. Cluster identities were defined after unconstrained integration with scRNA-seq profile. Peaks were called using MACS2^29^ (v2.2.9.1). We used the Peak matrix to identify Peak-to-gene links through “addPeak2GeneLinks” function, enabling further correlation analysis between peak accessibility and gene expression.

#### Identification of candidate TF regulators

ChromVar^30^, implemented in ArchR package, was used to predict enrichment of TF activity on a per-cell basis. To identify functional TFs across each cell type, Pearson correlation was calculated between the motif enrichment Z-score and the ATAC-inferred gene expression using the “correlateMatrices” function (FDR < 0.01 and correlation > 0.5). In addition, Pearson correlation analysis was also performed between ATAC-inferred gene expression and gene expression level from RNA-seq profiles.

#### Analysis of differentially accessible peaks

The processed ArchR project was converted to Signac “Seurat Object” format using the ArchRtoSignac package^31^ (v1.0.5) for differential accessibility analysis. Differentially accessible regions between the two treatment groups were determined by the “FindMarkers” function in the Signac package (v1.14.0), employing a logistic regression (L.R.) framework. Functional annotation of associated genes was performed using DAVID (v6.8, https://david.ncifcrf.gov).

#### Transcription factor footprinting and Regulatory Networks

Accessible peak regions of each cell type from different samples were called using MACS2 (v2.2.9.1) from within Signac (Signac::CallPeaks). TOBIAS^32^ was then used to infer TF activities through footprinting analysis on these differentially accessible peaks in both conditions – Bexarotene treated and Controls. TOBIAS is a command-line pipeline with multiple modules. The first step of the analysis is Tn5 insertion bias correction using the ATACorrect module. After removing the background Tn5 insertion cuts highlighting the effect of protein binding, ScoreBigWig was then used to calculate the footprint score. Next, we applied the BINDetect module to estimate differentially bound motifs between the two treatment groups based on scores, sequence, and motifs across major cell types. Each footprint site was assigned a log2FC (fold change) between two conditions, representing whether the binding site has larger/smaller TF footprint scores in comparison. Finally, based on the prediction of bound TFs, we used the CreateNetwork module to build TF regulatory networks. The resulted network was imported into Cytoscape (v3.10.3)^33^ for visualization.

#### ChIP-seq analysis and validation of snATAC-seq data

We analyzed FASTQ files derived from sequencing libraries of Bexarotene (n = 2) and Vehicle (n = 2) treated APP/PS1 mice at the same age and treatment schedule as those used for scRNA-seq and snATAC-seq experiments. Reads were aligned to the mouse reference genome (GRCm38) using Bowtie2 (v2.5.4) with the -local option. Low quality reads (mapping score < 2) were removed by Samtools (v1.21). Peak calling was performed with MACS2 at a *q*-value threshold of 0.05, comparing Bexarotene vs Vehicle groups. To validate genomic sites identified by TOBIAS BINDetect as enriched RXR motifs following Bexarotene treatment, we calculated the overlapping of those regions to the regions identified as enriched by ChIP-seq following Bexarotene treatment. Genomic coordinates of overlaps were determined by Bedtools (v2.31.1) with 10 kb genomic windows. The overlapping regions were annotated by Uropa with default options. The list of overlapping regions identified by ChIP-seq and ATAC-seq were further analyzed in Metascape^34^ for multilist comparative analysis, integration of the annotation information and enrichment analysis using GO processes, KEGG pathways, Reactome gene sets, canonical pathways, and CORUM complexes^35–39^. Overlapping peak sequences annotated to transcription factor genes were scanned using MEME Suite FIMO (v5.5.7) with default settings to identify candidate RXRα heterodimer motifs.

All data processing using R and python packages and command line tasks were conducted on RHEL Server equipped with Intel Platinum 8352M CPU, 1TB of RAM, 128 cores.

## Results

### Single-cell RNA and ATAC profiling identified cellular diversity within APP/PS1 mouse brain.

**Figure 1:**
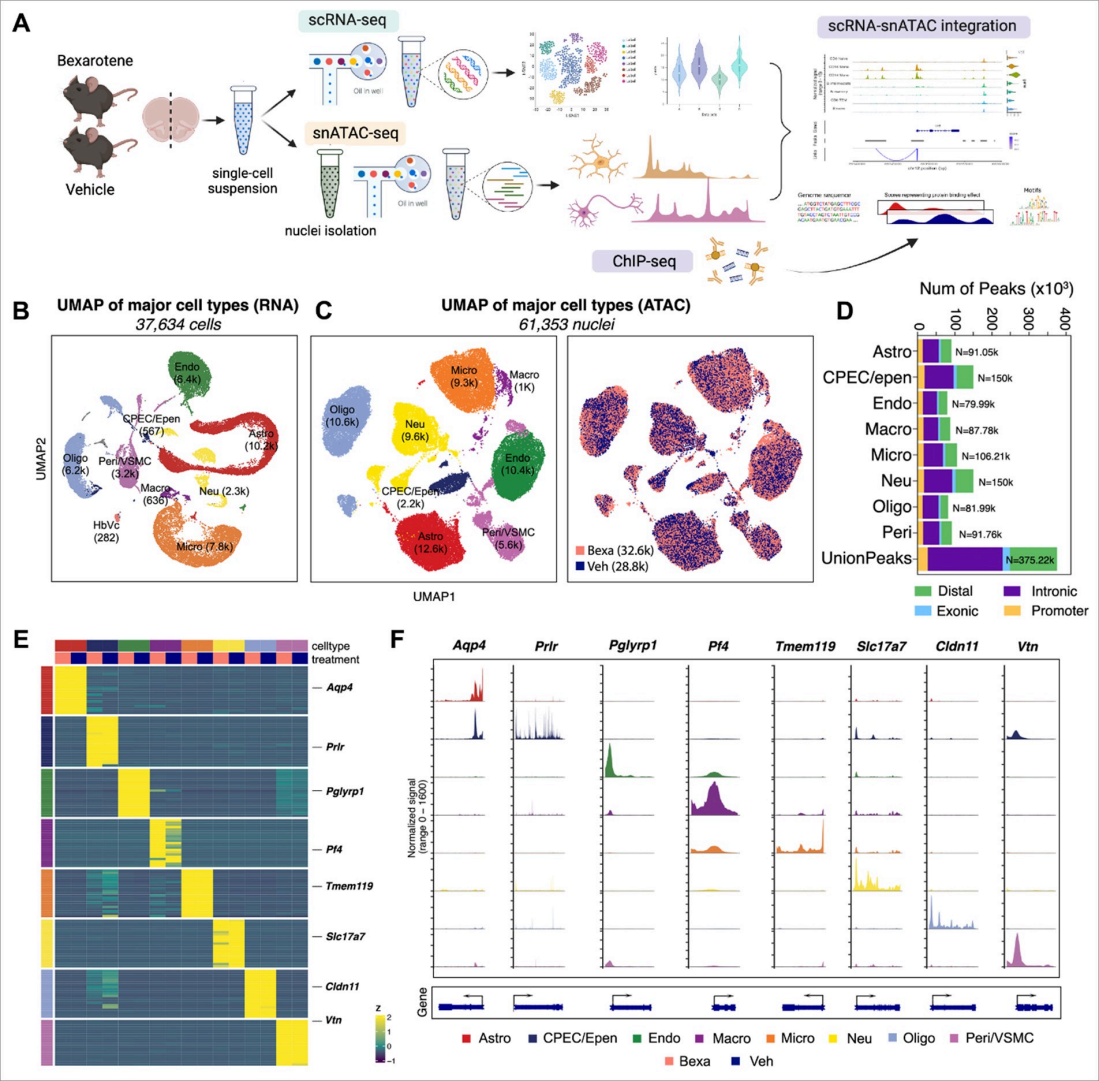
**(A)** Study design. APP/PS1 mice (5-month-old, male/female) were orally treated with bexarotene (Bexa, 100mg/kg/d) or vehicle (Veh, corn oil with 1% DMSO) for 10 days. Mouse brains were dissected and dissociated into single-cell suspension followed by scRNA-seq and snATAC-seq library preparation using 10x Chromium platform and sequencing. **(B)**UMAP projection of major cell-type clusters of scRNA-seq data with Seurat. **(C)**UMAP projections of sample treatment distribution across major cell-type clusters following integration of scRNA-seq and snATAC-seq datasets (Astro: astrocytes, CPEC/Epen: choroid plexus epithelial cells / ependymal cells, Endo: endothelial cells, Macro: macrophages, Micro: microglia, Neu: neuron, Oligo: oligodendrocytes, Peri/VSMC: pericytes / vascular smooth muscle cells). **(D)**Proportions of annotated peak distribution across major identified cell-type groups. n=5/group. **(E)** Heatmap for normalized gene expression of top features from each cell type. (F) Coverage plot showing chromatin accessibility across major identified cell types for cell-type specific marker genes.

In this study (Fig. 1A), we used 5-month-old APP/PS1 mice, an age known to develop early AD pathologies characterized by increasing amyloid plaque burden and gliosis^26^. Mice were randomized in two treatment groups for Bexarotene (Bexa, n = 5, 100mg/kg/day for 10 days) and Vehicle (Veh, n = 5, corn oil) as described in our previous studies^21,40^. Following treatment, mouse brains were dissected and dissociated using a protocol suggested by 10x Genomics, and the resulting cell or nuclei suspensions were processed on 10x Chromium platform for single-cell RNA or open chromatin profiling. Separate paired-end sequencing libraries were generated for single-cell RNA-seq (scRNA-seq, n = 8, 4 Bexa and 4 Veh) and single nuclei ATAC-seq (snATAC-seq, n = 10, 5 Bexa and 5 Veh). The sequencing reads were aligned to the mouse reference genome (GRCm38) and initially processed with Cell Ranger and Cell Ranger ATAC pipelines. The filtered feature matrix, BAM, and fragment files were further analyzed using Bioconductor packages Seurat, ArchR, and Signac (additional details are provided in the Methods and Supplemental material). We profiled transcriptional landscapes of 37,640 cells by scRNA-seq and analyzed 61,353 individual nuclei by snATAC-seq. While ArchR can robustly call clusters, it is not possible to know a priori which cell type is represented by each cluster. Because we apply ArchR to analyze chromatin architecture in brain following Bexa treatment, to perform cell type annotation we integrate the two library sets and use prior knowledge of cell type-specific marker genes. Figure 1B shows UMAP projection of major cell-type clusters derived by using scRNA-seq analysis; on Fig. 1C we present UMAP projections distributed across major cell-type clusters following integration of scRNA-seq and snATAC-seq datasets. Cells/nuclei from the two treatment groups projected into highly overlapping clusters (Fig. 1C). Major cell types identified in the two datasets include astrocytes, microglia, endothelial cells, macrophages, neurons, oligodendrocytes, pericytes, choroid plexus epithelial cells, and ependymal cells. Figure 1D shows the proportions of annotated peak distribution across major identified cell-type groups.

If multiple distinct samples are integrated in a single data set, it is important to compare various metrics across all samples. ArchR allows the identification of marker genes and plotting/visualization of those for a certain cell type and across the clusters. When analyzing chromatin accessibility based on ATAC-seq data sets, with ArchR it is possible to estimate gene expression from chromatin accessibility data by using calculated gene scores. A gene score predicts how highly expressed a gene will be based on the accessibility of regulatory elements in the vicinity of the gene.

Standalone ATAC-seq analysis can lead to the identification of peak co-accessibility and to predict regulatory interactions. Using a standard approach implemented in ArchR, we performed pairwise testing between cell groups and integrated multiple levels of information from scRNA-seq and snATAC-seq. On Fig. 1E we present normalized expression, obtained by scRNA-seq, of the top features across 8 major brain cell types divided into treatment groups. Major cell types identified by the enriched marker genes include astrocytes (*Aqp4*), endothelial cells (*Pglyrp1*), microglia (*Tmem119*), oligodendrocytes (*Cldn11*), neurons (*Slc17a7*), macrophages (*Pf4*), pericytes and vascular smooth muscle cells (VSMC, *Vtn*). There was one additional cluster containing choroid plexus epithelial cells and ependymal cells (enriched *Prlr*). Chromatin accessibility across most abundant cell types for cell type specific marker genes is presented graphically on Fig. 1F. We have been able also to identify so-called “peak-to-gene links.” Peak-to-gene linkage (Fig. 1F, bottom) is an advanced ArchR module to integrate scRNA-seq data and to look for correlations between peak accessibility and gene expression. Because peak-to-gene linkage correlates snATAC-seq and scRNA-seq data, these links are more relevant to gene regulatory interactions.

### Changes in chromatin accessibility in response to RXR ligand activation predict changes in expression of upstream regulators.

**Figure 2:**
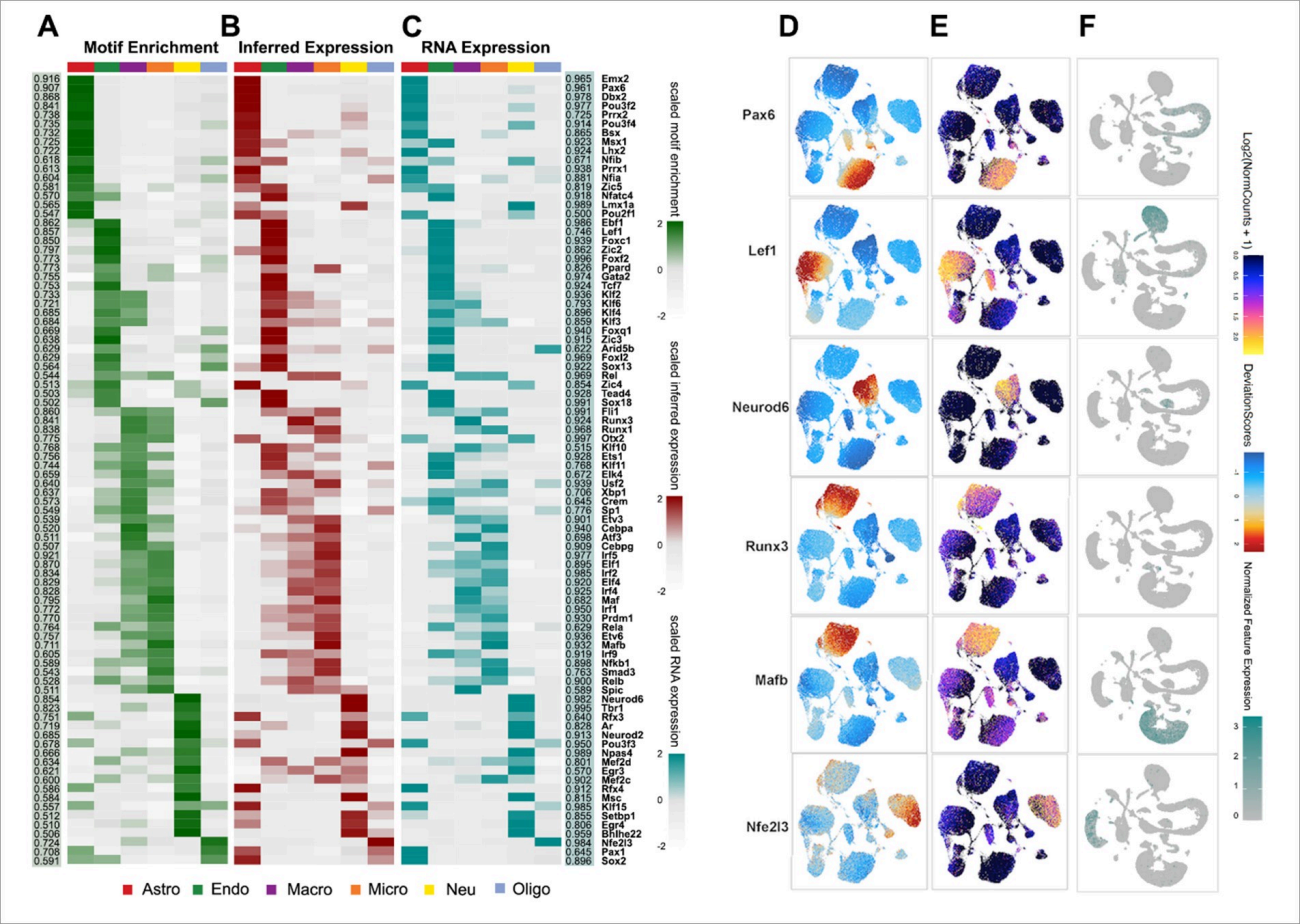
**(A-C)** Heatmaps of 88 candidate transcription factor (TF) regulators across major cell types shown as chromVar motif enrichment (A), inferred gene expression (B, ATAC gene-score from ArchR), and RNA expression level (C, from scRNA-seq). The two side panels show Pearson correlations between ATAC-inferred TF expression and chromVar (left, highlighted in green) or gene expression (right, highlighted in cyan). **(D-F)**UMAP embeddings of example candidate TF regulators as of motif enrichment (D) and inferred gene expression (E) from ATAC-seq (ArchR), as well as gene expression (F) from RNA profile (Seurat). The examples of cell-specific TF regulators are: *Pax6* in astrocytes, *Lef1* in endothelial cells, *Neurod6in* neurons, *Runx3* in macrophages, *Mafb* in microglia, and *Nfe2l3* in oligodendrocytes.

To systemically identify potential transcriptional regulators shaping the chromatin accessibility landscape of brain cells, we performed motif enrichment analysis using the “chromVar” function integrated in the ArchR toolkit. This approach quantified TF motif enrichment in accessible chromatin regions across major cell types following RXR ligand activation (Fig. 2A–C). Specifically, it measures how frequently certain motifs are found in genomic regions, such as those upstream of TSS, where regulatory elements are often located. To validate our predictions and prioritize functionally relevant transcriptional regulators, we also examined their expressions and correlations with motif enrichment across cells (Fig. 2B). We discovered 88 high-confidence candidate regulators that show strong correlations between enrichment of TF motifs and ATAC-inferred expression, as well as between ATAC-inferred expression and RNA-measured expression (Pearson > 0.5, false discovery rate [FDR] < 0.01, Table S1). These include cell-specific TF such as: *Pax6* in astrocytes, *Lef1* in endothelial cells, *Neurod6* in neurons, *Runx3* in macrophages, *Mafb* in microglia, and *Nfe2l3* in oligodendrocytes (Fig. 2D–F).

### Bexarotene treatment induces cell-type specific epigenomic changes in APP/PS1 mouse brain.

**Figure 3:**
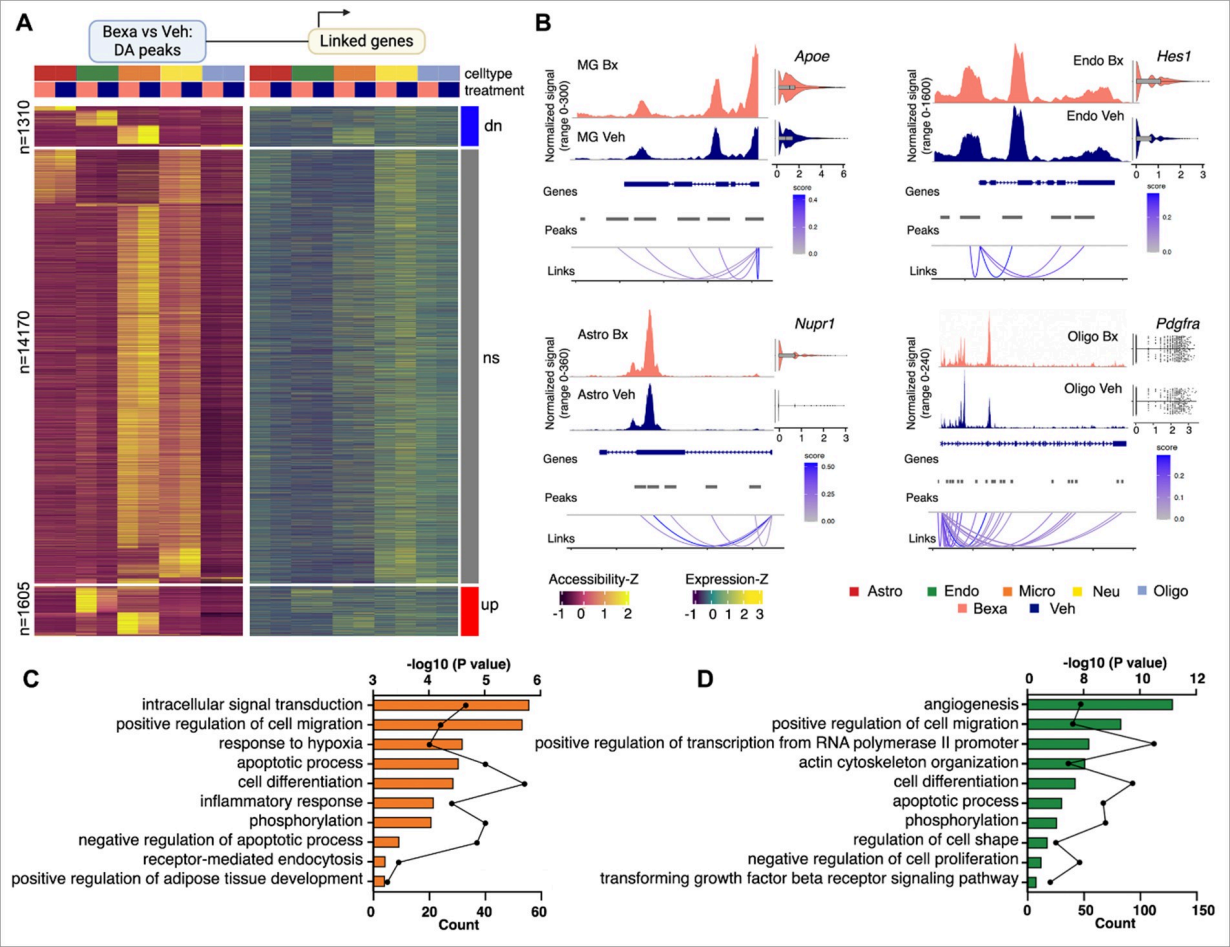
**(A)** Heatmap of differentially accessible peaks (left) and their linked genes (right) between Bexa- and Veh-treated groups across major cell types. **(B)** Coverage plot of differentially accessible peaks in various cell types, including gene annotations, peak coordinates, genomic links and gene expression levels in Bexa- and Veh-treated groups. **(C-D)** Biological process associated with more accessible peaks and upregulated genes in Bexa-treated microglia (C) and endothelial cells (D).

Next, we used the integrated single cell RNA-seq and ATAC-seq data to explore cell-type- and disease-specific cis-regulatory elements (CREs) and their target genes following different treatment. The Cell Ranger ATAC pipeline produces the correlations as “feature linkages”. A feature linkage, or simply link, is defined as a significant correlation between accessibility of an ATAC peak and the expression of a gene. We restricted this correlation analysis to consider only peaks within 50–100 kb of each TSS. We first took the union of ATAC peaks identified in each cell type and retained only those present in over 2% of cells in at least one cell type. Most peaks have similar number of counts across the 5 major cell types in Bexa and Veh treated groups (Fig. 3A **and Table S2**, shown as “ns” in the middle of the heatmap, n = 14,170). However, we identified 1,605 peaks that are more accessible in the Bexa-treated group, and 1,310 peak signals enriched in the Veh-treated group (Fig. 3A). These differentially accessible peaks mostly arise from microglia and endothelial clusters. We then asked if the differential gene expression we observed could be mediated by candidate CREs. The majority of differentially expressed genes (DEGs) identified between Bexa and Veh treated nuclei had a linked peak in the same cell type where the gene was differentially expressed. An example is the *Apoe* gene located in the *Apoe/Apoc* locus that was significantly upregulated in microglia of Bexa treated samples (log2FC = 0.42, adj-p = 3.23×10^−6^), in oligodendrocytes of the same group (Log2FC = 0.39; adj-p = 2.02×10^−51^) but not in astrocytes. As shown on Fig. 3B, several peaks close to the TSS are higher in Bexa treated microglia. Other examples are *Hes1* in endothelial cells (Log2FC = 0.70; adj-p = 2.73´10^−8^), *Nupr1* in astrocytes (Log2FC = 0.52; adj-p = 6.28´10^−4^), and *Pdgfra* in oligodendrocytes (Log2FC = 0.72; adj-p = 4.96´10^−4^). Considering most of the differentially accessible peaks are in microglia and endothelial cells, we then did gene ontology (GO) enrichment analysis using lists of associated genes (Fig. 3C–D, **Table S2**). Consistent with the GO enrichment analysis of transcriptome profiling, chromatin profiles indicated also that Bexa treatment triggered developmental pathways in both cell types including cell proliferation, differentiation and migration, apoptotic process, and phosphorylation. In addition, angiogenesis and inflammatory response were upregulated in endothelial (Fig. 3D) and microglia (Fig. 3C) cluster, respectively.

### Bexarotene-activated RXR induce cell-type specific transcriptomic changes.

**Figure 4:**
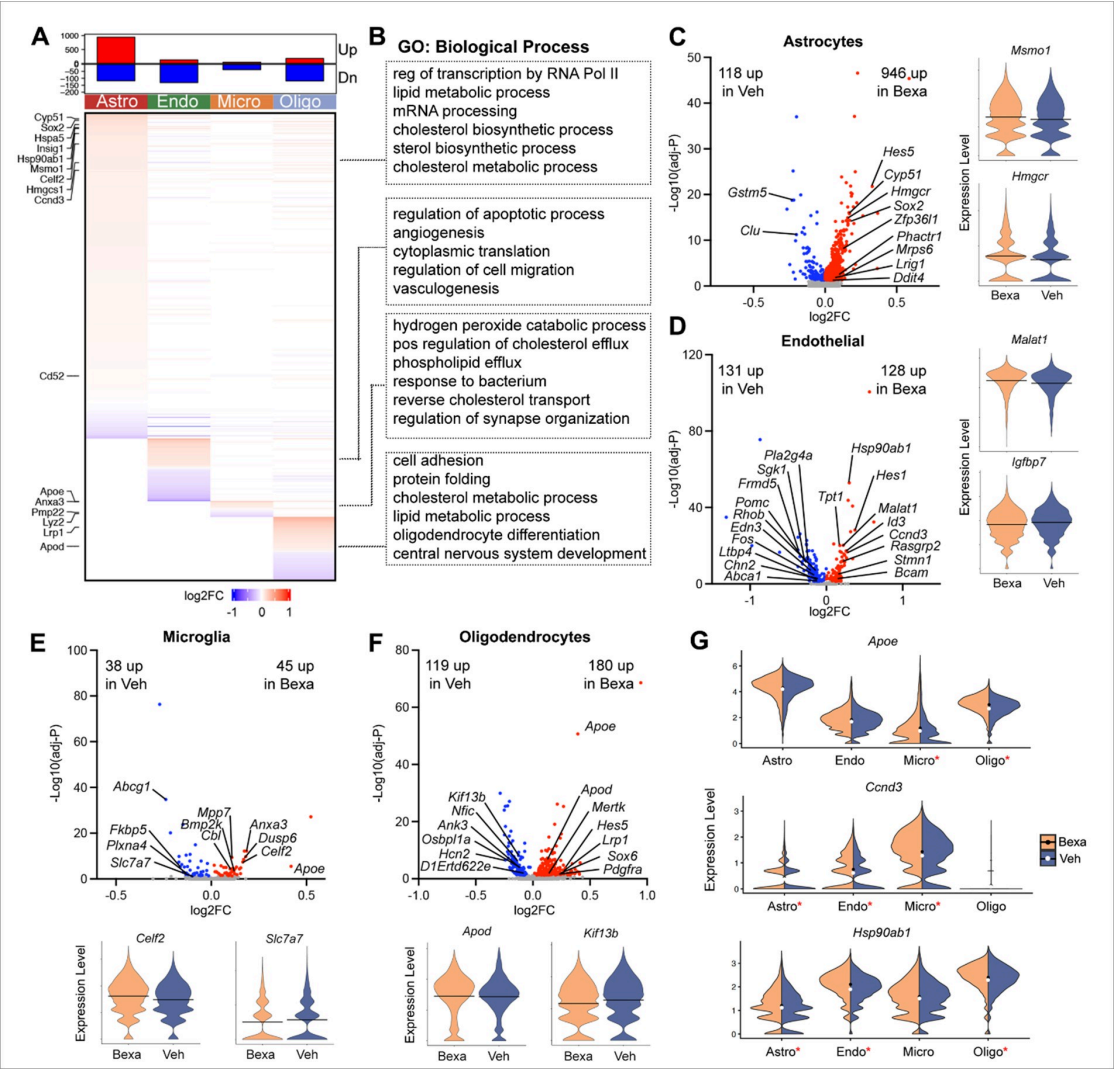
**(A)** Heatmap showing MAST-adjusted log2FC of cell type-specific differentially expressed genes (DEGs) in response to Bexa treatment. The top panel indicates the number of DEGs in each cell type. **(B)**Gene Ontology (GO) enrichment analysis of biological processes associated with DEGs from each cell type. **(C-F)** Volcano plots show DEGs, with upregulated genes in red and downregulated genes in blue following Bexa treatment, from the four-cell-type of interest: astrocytes (C), endothelial cells (D), microglia (E), and oligodendrocytes (F). Accompanying violin plots illustrate expression of two representative DEGs per cell type. Crossbars on the violins indicate mean expression level for each group. **(G)** Violin plots showing normalized expression level of *Apoe*, *Ccnd3*, and *Hsp90ab1* across the four cell types, split by treatment groups. Black dots denote mean expression levels for Bexa-treated group; white dots denote Veh controls. Asterisks (*) indicate significance (adjusted p-value < 0.05) within cell types.

We next examined changes in transcriptome profiles in different cell types following Bexa or Veh treatment. A total of 1,689 genes across the four cell types of interest were identified as significant, differentially expressed and many of them were cell-type specific (Fig. 4A **and Table S3**). Astrocytes exhibited the most DEGs, having 946 genes significantly up- and 118 genes down-regulated. Genes involved in multiple metabolic processes (Fig. 4B), including lipid, sterol, and cholesterol metabolism, such as *Msmo1*, *Insig1*, *Hmgcr*, *Hmgcs1*, *Cyp51*, and *Idi1* were found significantly upregulated in astrocytes (Fig. 4C). Upregulation of SRY-box transcription factors (Sox) genes (*Sox2*, *Sox9*, *Sox21*, etc.), cyclin family genes (*Ccnt2* and *Ccnd3*, Fig. 4G), and Hes family bHLH transcription factor genes (*Hes5* and *Hes1*) led to elevated developmental pathways in astrocytes, endothelial cells, like the regulation of transcription and translation, and cell differentiation and proliferation. Genes of the heat shock protein family - *Hspa5*, *Hspa8*, and *Hsp90ab1*, which are key players in pathways including protein folding and stabilization^41^, showed increased expression levels in astrocytes, oligodendrocytes, and endothelial cells (Fig. 4G). Lipid-associated metabolic processes and developmental pathways were also seen to be upregulated with Bexa treatment in oligodendrocytes (Fig. 4F). Genes that contribute to those pathways include *Lrp1*, which encodes a low-density lipoprotein receptor protein^42^, and *Apod*, a critical mediator of lipid transport, remyelination, and nerve regeneration^43^. As compared to the differential accessibility results described in the previous section, endothelial cells (Fig. 4D) exhibit the most overlapping genes between the DEG and DA lists, with 23 genes upregulated and 18 downregulated in both lists following Bexa treatment. For example, *Hes1* (Fig. 3B) and *Ccnd3* (Fig. 4D) are upregulated in endothelial cluster in both analyses. In microglia, Bexa treatment induced upregulation of some disease associated microglia (DAM)^44^ signatures like *Apoe* and *Anxa3*, contributing to the upregulation of inflammatory responses (Fig. 4E). *Celf2*, a key player in controlling the splicing of multiple microglial features, was upregulated in microglia as well (Fig. 4E). On the other hand, some DAM signatures, such as *Cd52*, *Lyz2*, and *Pmp22*, were downregulated in microglia. Of note, *Apoe* was upregulated in all cell types with Bexa treatment (Fig. 4G), but only showed significance in microglia and oligodendrocytes, with the highest fold change achieved in microglia. It is known that *Apoe* is an RXR target gene and one of the DAM signatures, likely under the control of LXR/RXR heterodimer activation^20^.

### ATAC-seq footprinting unravels the complexity of RXR transcription factor binding and a network of trans-regulatory interactions.

**Figure 5:**
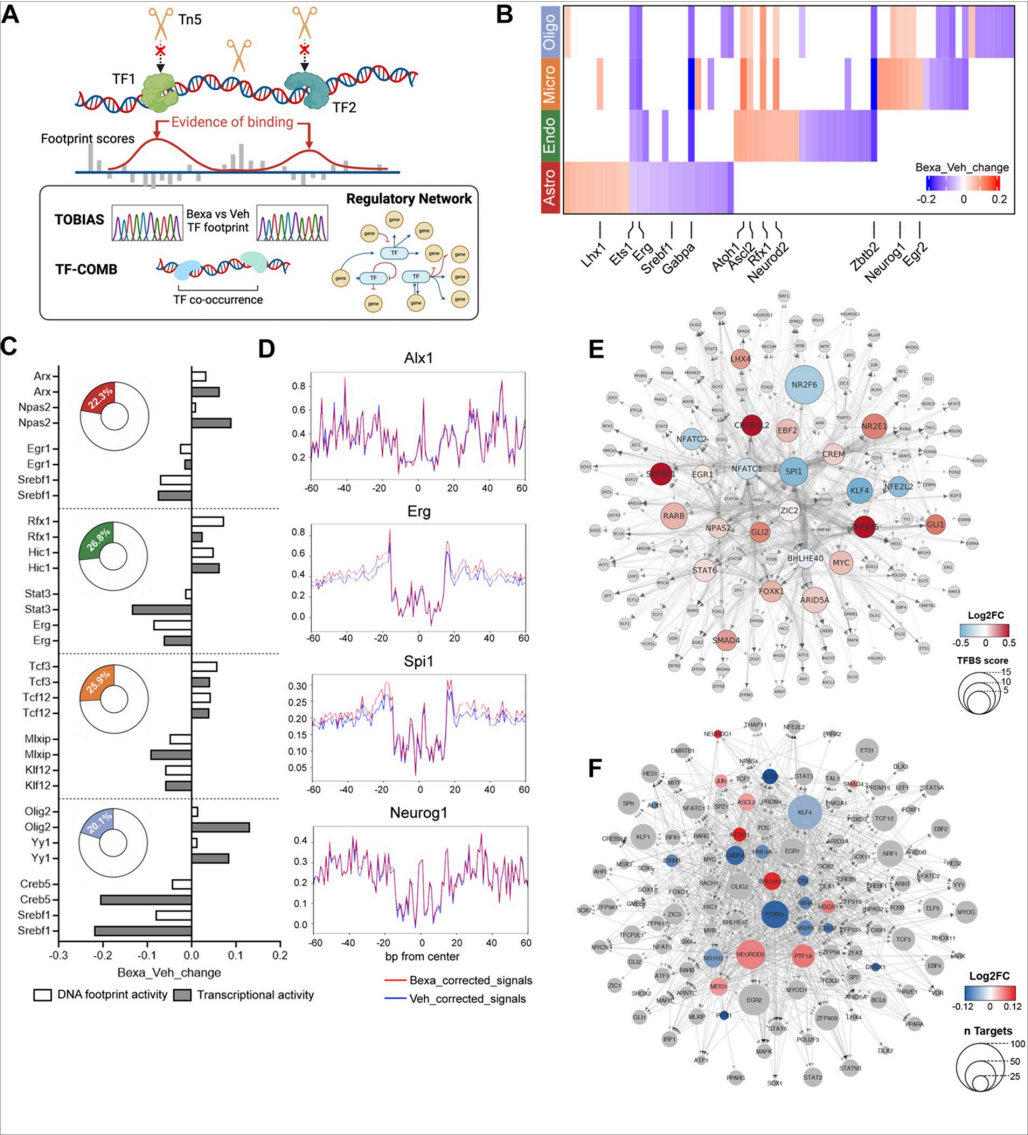
**(A)** Schematic workflow for transcription factor (TF) footprinting, regulatory network, and co-occurrence analysis. **(B)**Heatmap of differentially bound TFs between Bexa and Veh treated groups across astrocytes, endothelial cells, microglia, and oligodendrocytes. Red indicates a higher score of Bexa_bound; blue indicates higher Veh_bound. **(C)** Bar charts showing representative TF occupancy and expression changes (log2 FC, Bexa versus Veh) in open chromatin regions (white bars) and transcriptional activity (grey bars) for each cell type. Donut plots present the percentage of TFs with concordant changes in occupancy and transcriptional activity. **(D)**Representative footprints of significantly (FDR > 0.05) changed TF occupancy in open chromatin: Alx1 in astrocytes, Erg in endothelial cells, Spi1 in microglia, and Neurog1 in oligodendrocytes. Red lines represent corrected signals in Bexa-treated cells; blue lines for Veh-treated cells. **(E)** TF regulatory network of RXRa. The TF-TF network is constructed from all TFBS bound in TF promoters associated with RXRa. Node shapes represent the hierarchical levels in the network starting from RXRa (circular: TFs directly regulated by RXRa octagons: the TFs indirectly regulated by RXRa — targets of its direct TFs). For the circles (direct TFs), node color indicates mean log2FC in binding score (Bexa versus Veh); node size represents the TFBS score. **(F)** Neuronal TF regulatory network of transcription factors with significantly different binding scores (Bexa versus Veh). Colored circles represent transcription factors (TFs) with significantly different binding scores (Bexa versus Veh). Gray circles represent the direct target TFs of these significantly changed TFs. The size of each circle indicates the number of direct TF targets for each TF, and the color represents the mean log_2_ fold change (log_2_FC) in binding score (Bexa versus Veh).

To further explore changes in regulation of transcription in response to Bexa activated RXR in APP/PS1 mouse brain and to assess relevant changes in TF binding we analyzed the open chromatin profiles of four major brain cell types – astrocytes, endothelial cells, oligodendrocytes, and microglia (Fig. 5). We analyzed the TF binding activity using TOBIAS (Fig. 5A). TOBIAS is а computational genomic footprinting framework that enables genome-wide investigation of TF binding dynamics for hundreds of TFs simultaneously^32^. Genomic footprinting is a molecular biology technique used to identify specific regions of DNA that interact with TFs or other proteins within a genome. These interactions leave “footprints” – regions of DNA that are protected from being cleaved or chemically modified. TOBIAS allows identification of footprints and analyzes enrichment/abundance of peaks overlapping with TF binding motifs. Thus, DNA footprints overlapping with TF motifs are defined as direct TF binding sites. TOBIAS calculates and presents the distance between the motifs and the TSS, genomic structures and features in both directions, and is a powerful tool to construct TF regulatory networks^32^.

We used ATACorrect module of TOBIAS to correct for the inherent Tn5 transposase insertion bias. The module also corrects for false positive footprints in uncorrected signals and identifies TFs for which the motif itself disfavors Tn5 integration. Thus, ATACorrect quantifies TF binding activities by scoring the depleted signal at each footprint’s predicted binding site relative to the surrounding background chromatin accessibility. This bias correction effectively uncovers TF footprints, which were otherwise superimposed by Tn5 bias.

We used BINDetect module on integrated data sources from a set of samples to identify TFs with predicted significant changes in binding between the two treatment groups. The top TFs with −log10(p-value) above the 95% quantile and/or differential binding scores smaller/larger than the 5% and 95% quantiles (top 5% in each direction) were considered significant and are illustrated in the heatmap in Fig. 5B and **Figure S2**. Some TFs, such as *Ets1*, *Erg*, and *Gabpa*, showed higher binding scores in the control group across all four cell types. *Neurod2*, another example, showed higher binding score in endothelial cells, microglia, and oligodendrocytes. In contrast, the cell type-specific TF responses to the treatment were more prominent in astrocytes. Comparing the TF binding changes to the transcriptional activity (shown in Fig. 4), we found that over 20% (20.1% − 26.8%) of the changes in DNA footprinting following Bexa treatment were consistent with the corresponding gene expression changes (Fig. 5C and **Table S4**). For example, in oligodendrocytes and astrocytes, Olig2 had higher binding scores in the treatment group (astro-foot-log2FC = 0.02, oligo-foot-log2FC = 0.01) and also showed higher expression (astro-DEG-log2FC = 0.09, oligo-DEG-log2FC = 0.13) in the treatment versus control groups (Fig. 5C, **Table S4**). Figure 5D presents examples of altered footprints for Alx1 in astrocytes, Erg in endothelial cells, Spi1 in microglia, and Neurog1 in oligodendrocytes.

Since the RXR and the permissive RXR heterodimers are the primary targets of Bexarotene, not surprisingly most of the predicted RXR targets encode TFs themselves. Therefore, in the next step, we applied the TOBIAS Network module to further match enriched binding sites to TF targets with the aim of revealing how these TF activities might connect. We used the combined pool of binding sites to investigate and construct TF binding networks comparing the two treatment groups, Bexa and Veh, regardless of the cell types (Fig. 5E). Thus, we were able to build a directed network and to predict a TF activation cascade initiated by ligand activated RXR. This network resulted in the predicted activation of 25 primary TFs which subsequently activate 141 additional TFs.

### Differential Regulatory Networks in Bexa-Treated Neuronal Systems

As already noticed in the introduction, the most pronounced therapeutic effect of Bexarotene in APP expressing mice, or other models of neurodegeneration, confirmed in multiple labs, has been on behavior and brain inflammatory response. That was one reason to conduct and present the footprinting and TF networking results for neurons separately. Collectively, the analysis of neuronal TF regulatory networks in Bexa-treated versus vehicle-treated (Control) groups reveals a system characterized by overall structural robustness with distinct shifts in localized TF influence. Despite starting our pathfinding from differentially bound TFs revealed by BINDetect (Meis1, Atoh1, Neurod2 for Treated; Foxn1, RXRa for Control), the overarching network architecture, encompassing 134 TFs and thousands of edges, remains remarkably consistent in size, density (≈ 0.18 − 0.20), and global connectivity (Fig. 5F). This suggests that Bexarotene doesn’t dismantle the fundamental regulatory scaffold in neurons but rather modulates its activity through specific node.

A consistent core of universal master regulators—notably Klf4, Egr2, Zfx, Foxn1, and Nrf1—emerges with the highest out-degrees across all conditions, forming an identical, broadly radiating subgraph irrespective of the initial seed TF. This underscores their persistent, foundational role in regulating the broader TF landscape in both treated and control states.

In essence, Bexarotene treatment appears to selectively enhance specific regulatory cascades (Atoh1 → Neurod2) and activate highly focused, direct regulatory hubs (Neurod2 itself). Conversely, the control TFs delineate the baseline regulatory landscape, featuring broad cascade initiators (Foxn1) and crucial signal integrators (RXRa). The observed differential binding, therefore, translates into distinct topological changes in how regulatory signals are initiated, propagated, and integrated, offering specific hypotheses for Bexarotene’s mechanism of action at the network level.

### ChIP-seq validation of snATAC-seq peaks.

**Figure 6:**
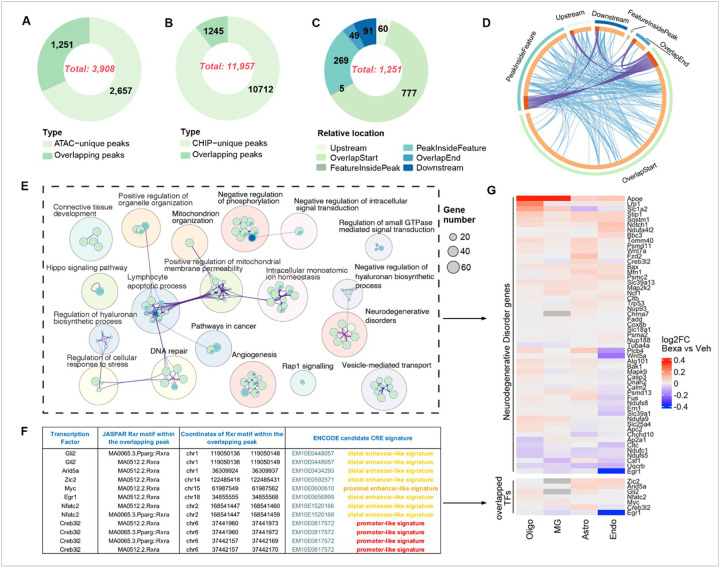
**(A-C)** Numbers of unique and overlapping peaks identified by ATAC-seq and ChIP-seq and their relative location in predicted regulatory regions of the nearest genes. **(D)** Circos plot visualizing the overlap among the gene lists. The outside arcs represent the identity of each gene list. On the inside, each gene member of the corresponding list is assigned a spot on the arc. The dark orange color represents the genes shared by multiple lists, and the light orange color represents genes unique to that gene list. Purple lines link the same genes that are shared by numerous gene lists. Blue lines link the genes under the same statistically significant, enriched ontology term. **(E)** Network of enriched signaling pathways of genes with overlapping ATAC-seq and ChIP-seq peaks. The dot size corresponds to the number of genes enriched in the corresponding pathways. The sector area within the circle (pathway(s)) represents the proportion of enriched genes with overlapping peaks of the corresponding relative location, as shown in the inset. The annotated nearest genes were processed in Metascape (http://metascape.org) for GO and pathway enrichment analyses. The results were visualized with Cytoscape. **(F)** The columns in the Table are TF names, JASPAR RXRa (PPARg:: RXRa) motifs and their coordinates within the overlapping ATAC-seq and ChIP-seq peaks and ENCODE cCRE signatures. Since Chip-seq assay was conducted with RXR antibody, only predicted RXRa homo- and heterodimers are included in the Table. See details in the Methods. **(G)** In the heatmap the expression level of the nearest to the overlapping peaks genes involved in neurodegeneration is presented as log2FC for each of the cell types. The lower panel is the expression level of all TFs by cell type identified as genes near to ATAC-seq and ChIP-seq overlapping peaks.

We performed ChIP-seq with RXR antibody^21,22^ using brain tissue from APP/PS1 mice, and treatment schedule as already described for scRNA-seq and snATAC-seq assays. We applied MACS2 peak calling tool^29^ and identified 11,957 significant peaks in Bexarotene vs Control treated mice (Fig. 6B). We used 3,908 significantly enriched ATAC-seq peaks (Fig. 6A) to filter out the overlapping ones and to compute their relative location to the nearest Features (Fig. 6C). Out of 1,251 overlapping peaks 777 were covering TSS of the annotated genes. Adding 60 overlapping peaks upstream of the TSS, it is reasonable to assume that almost 70% of the overlapping ATAC-seq/ChIP-seq peaks are proximal CREs, located upstream of TSS, that contribute to or control the expression of the nearest genes in case RXRs are ligand stimulated. The Circos plot on Fig. 6D is presenting the overlap among the gene lists at corresponding locations of RXR antibody bindings (ChIP-seq) and enriched RXR motifs (ATAC-seq) and gives an idea of the complexity of enrichment of GO terms affected by the treatment of RXR synthetic agonist Bexarotene. The results of GO enrichment and pathway analyses using 1,251 overlapping annotated peaks over gene structures with predicted regulatory function are presented on Fig. 6E. The network as shown is built of predicted GO terms and pathways presumably affected and to some extent, controlled by ligand activated RXR or rather RXR heterodimers. Their binding to cis-regulatory elements has been validated by ATAC-seq and ChIP-seq assays. It is important to notice that not all genes of the network are directly controlled by ligand activated RXR. While this phenomenon is well known, if the network is a result of overexpression or ligand activation of TF, in most of the cases it is not possible to identify the direct regulator of some of transcriptionally upregulated genes. The integration of ATAC-seq derived TF footprinting, ChIP-seq and scRNA-seq data provides such an opportunity. We demonstrate this by further analyzing the set of genes with overlapping peaks comprising KEGG pathways associated with neurodegenerative disorders – AD, Parkinson’s disease (PD), Huntington’s disease (HD), Prion disease (PrD) and Amyotrophic Lateral Sclerosis (ALS). First, these genes have been selected as the nearest ones after the annotation – structural and functional, out of all overlapping peaks and processed for GO pathway enrichment analysis by the software package Metascape in completely unbiased way. Second, since we already had scRNA-seq conducted, we filtered out all genes associated with neurodegenerative disorders – AD, PD, HD, PrD and ALS, as found in the first step, and in Fig. 6G we present their differential expression in all four cell types. The results of these analyses demonstrate that the multiomics approach to study changes in phenotypes in response to ligand-activated retinoid nuclear receptors presents an exclusive opportunity to reveal changes in gene expression associated with distinct changes in chromatin accessibility as a result of cascades of TF binding events (see also Fig. 5 and the previous section).

### The multiomics analysis of RXR ligand activation is a powerful approach to assign RXRs to candidate CREs

For the last step of our multiomics integrative analysis, from the list of annotated overlapping ATAC-seq and ChIP-seq peaks (**Table S6**), we selected only annotated TFs and addressed biologically rich, but still incompletely understood space – TF occupancy by RXR homo- or heterodimers within ENCODE candidate CRE (cCRE) of the nearest genes. According to a study published by Neph et al.^45^ ~50-bp chromatin structure upstream from GENCODE TSSs is a highly stereotyped footprint comprising a prominent central DNase I footprint flanked symmetrically by 15-bp regions of uniformly elevated levels of DNase I cleavage. Based on that, a 35–55-bp footprint was considered the predominant feature of many promoter DNase hypersensitive sites^47^ and is in tight spatial coordination with the TSS^47^. However, how close or far from a TSS should a cCRE be to initiate or enhance the assembling of the transcriptional machinery and efficient start of transcription is not so clear. The UCSC genome browser (UCSC GB) presents cCREs, essentially, within each annotated gene structure. We used ENCODE conventions and configuration definitions of regulatory signatures to determine if and where the overlapping motifs within those signatures are located. On **Table S6** we list, and on Fig. 6G we show changes in expression by log2FC of seven annotated TFs as genes nearest to the overlapping peaks, the genomic coordinates and ENCODE signatures within the peaks adjacent to those TFs. In a second step using MEME tools we identified TF motifs within the overlapping peaks. Since the ChIP-seq validation of ATAC-seq peaks was performed using RXR antibody, from the output FIMO tables we chose only the coordinates of RXRa or Pparg::RXRa motifs. It is an important clarification, because the scanning of DNA sequences within overlapping peaks against a database of canonical motifs will create a table of motifs with predicted TFs. Some of them will be presented with binding affinities higher than the affinity of RXRa. Moreover, some of those TFs are known to exert their transcriptional regulatory power as obligate RXRa heterodimers. Under such stringent criteria used to assign a TF to a CREs near a gene, we are obviously losing cCREs at a distance of 10kb up- and down-stream from the TSS – an approach applied in many ChIP-seq studies^48^. Our results demonstrate that RXRa presumably as a homo/heterodimer^5^, was bound to an ENCODE classification signatures, with an impact on the corresponding/nearest gene/TF expression (Fig. 6G, lower panel). Moreover, the expression level of the TFs is obviously cell type specific, which clearly indicates a modulatory effect of transcriptional coregulatory proteins engaged in interactions with RXRa, or effects of other TFs bound to CREs in a close vicinity or far from RXR response elements.

We systematically analyzed transcriptional regulators for TFs *Myc*, *Arid5a*, *Creb3l2*, *Egr1*, *Zic2*, *Gli2*, and *Nfatc2* as genes with overlapping ATAC-seq and ChIP-seq peaks within ENCODE regulatory signatures. We performed the analyses in the context of their function and predicted RXRa transcriptional regulation. These TFs are highly relevant to cell signaling, development, immune responses neurological functions and disorders. Each of those exhibits a distinct regulatory architecture, reflecting its functional specialization.

*Arid5a* was predominantly associated with pro-inflammatory transcription factors including STAT3, NF-kB, and AP-1^46,47^. Regulatory factors for *Creb3l2* included *ATF4*, *XBP1*, and *CEBPβ*, consistent with its activation under ER stress conditions and acute phase response^48^. Analysis of *Egr1* revealed upstream control by SRF, ELK1, and CREB, all of which are involved in activity-dependent gene expression, lung disease and response to infection^49,50^. Moreover, Egr1 has been revealed as a major mediator and regulator of synaptic plasticity and neuronal activity in both physiological and pathological conditions^51,52^. *Zic2* is controlled by developmental TFs such as OTX2, SOX2, and LHX2^53^, while Gli2 was associated with canonical Hedgehog pathway mediators GLI1, SUFU, and FOXC1^54^. None of the above TFs has been reported as transcriptionally controlled by RXR.

Of the seven TFs analyzed, only *Myc* and *Nfatc2* showed already known direct regulatory input from RAR (RXRa/RAR heterodimer), highlighting a limited yet targeted involvement of retinoid signaling in their transcriptional networks. In addition to RAR, *Myc* was found to be regulated by MAX and SP1, suggesting a role for retinoid signaling in modulating *Myc* expression. *Nfatc2*, a key immune regulator, was found to be transcriptionally regulated by NFAT, AP-1, and notably RAR, indicating responsiveness to both calcium and retinoic acid signaling. There are no studies about *Myc* or *Nfatc2* based on ChIP-seq validation assays and thus directly implicating RXRs in their transcriptional regulation. Based on our results, in addition to direct DNA binding of RXRa demonstrated by ChIP-seq assay, it is possible that a functional interaction of SP1 and RXRa is involved in the transcriptional control of *Myc*. The transcriptional control of *Nfatc2* seems more complicated. It has been demonstrated that NFATc2 directly bound to RARa and RXRa which enhanced the binding of RARa to the RA response element half-site^55^ – a well-defined phenomenon of RXRa heterodimerization and its binding to response elements. We consider the RXR transcriptional regulatory network and cooccurrence of RXRa and other TFs as an independent validation of those bindings. As shown in the previous section, *Nfatc2* is directly regulated by RXRa **(Table S6)** and binding of RXRa/NFATc2 pair significantly changes in response to Bexarotene treatment. The exact functional and physical interactions of RARa/RXRa heterodimers and NFATc2 as well as the transcriptional control of *Nfatc2* by RXRa and RXRa/RARa heterodimers in the context of immune regulation, development of numerous mammalian cell types and cellular adaptation^56^ requires further investigation.

## Discussion

In this study, we employed an integrated multi-omics approach, including scRNA-seq, snATAC-seq, and ChIP-seq, to investigate the effects of Bexarotene treatment in an AD-like mouse model. By combining transcriptomic and chromatin accessibility profiling at single-cell resolution with TF binding validation by ChIP-seq, we elucidated the regulatory landscape mediated by ligand-activated RXR in brain.

Single-cell RNA sequencing revealed cell-type-specific gene expression responses to Bexarotene, with notable enrichment in pathways related to developmental processes and lipid metabolism. Upregulated genes included members of the cyclin, Hes, and heat shock protein families, all known to contribute to developmental regulation. Additionally, genes involved in lipid metabolic pathways were broadly upregulated across cell types. In microglia, Bexarotene specifically enhanced biological processes such as reverse cholesterol transport and phospholipid efflux—key components in high-density lipoprotein (HDL) formation and lipid clearance.

Integration of snATAC-seq with transcriptomic data and cell-type annotation enabled the identification of differentially accessible chromatin regions and active TFs in a cell-type-specific manner. Microglia and endothelial cells showed the most prominent changes, particularly in regulatory regions associated with metabolism and development. Beyond global accessibility shifts, ATAC-seq footprinting revealed insights into TF binding activity. Leveraging recently developed R and Python-based tools for TF footprinting, we mapped the activity of RXR and its heterodimeric partners, uncovering distinct cell-type-specific regulatory networks. The results of this study clearly confirm that the obligate heterodimerization of RXRA with other NRs – NR1H3 and PPARG, is a cornerstone of nuclear receptor biology and has a significant transcriptional regulatory role in brain of AD model mice. RXR/NR1H3 dimerization is not a speculative interaction but a fundamental mechanism. The differential enrichment in the control group suggests that in the baseline, Control state, NR1H3/RXRA activity (perhaps driven by endogenous ligands or baseline cellular conditions) is a significant regulator, and Bexa treatment then shifts the regulatory landscape to favor other TFs or pathways.

The implications of this study extend beyond documenting chromatin and transcriptional changes in response to Bexarotene. Our approach demonstrates that integrating scRNA-seq, snATAC-seq, and ChIP-seq provides a powerful framework to construct high-resolution TF regulatory networks. These networks offer valuable insight into how ligand-activated TFs orchestrate gene expression programs in a tissue- and cell-type-specific context. Importantly, this strategy reveals how linear signaling pathways are converted into complex regulatory cascades through hierarchical TF interactions—bridging mechanistic studies with translational potential. Although RXR is a nuclear receptor, the outlined protocol is broadly applicable to other TFs, provided their activation context - be it natural, physiological, or pharmacological, is well-defined.

## Conclusions

We demonstrate that the integration of scRNA-seq, ATAC-seq, and ChIP-seq enables the simultaneous analysis of transcriptomic and epigenomic changes in individual brain cells. This approach offers a robust platform for dissecting the complex regulatory mechanisms of RXR in the central nervous system. Our findings provide new insights into brain cell-type-specific responses to RXR agonists and highlight the therapeutic potential of targeting nuclear receptors in Alzheimer’s disease.

## Limitations of the Study

We do not provide a direct demonstration that changes in TF networks following Bexarotene treatment of APP/PS1 mice are associated with changes in their phenotype. As we mentioned in the previous sections, numerous previously published studies, including this laboratory, confirmed that Bexarotene treatment, based on variety of cognitive tests, leads to improvement in cognitive performance. Those have been robust and reproducible studies. Considering the volume and the complexity of the analyses performed in this study we decided not to replicate validations by behavioral tests or changes in inflammatory response.

Changes in cognitive performance or response to inflammatory stimuli are not the only changes that can be further investigated in AD model mice. We can’t anticipate which one of tens of TF regulatory networks in the major brain cell types would be the most important or the most prospective from a therapeutic point of view in major neurodegenerative disorders. That is one reason we focused on TF regulations in neuronal systems. The results of our study, however, provide a rich resource to start the generation of “Atlas of RXR regulated TF networks in brain”. Since Bexarotene is an FDA approved cancer treatment drug, obviously there is a wide-open fieldto address and reveal the molecular mechanisms underlining the known and specific therapeutic effect of the drug.

For this study, the amount of text, the number of graphs and tables, necessary to present, restrict the incorporation of TF RXR co-occurrence (with other TFs). We realize the importance of such a Result section in the manuscript that would have had a significant contribution towards a complete list of RXR heterodimers in major brain cell types that have an indispensable role in brain development and neurodegeneration. All this is a matter of another manuscript, submitted elsewhere.

## Supplementary Material

Supplementary Files

This is a list of supplementary files associated with this preprint. Click to download.

- tableS1chromVar.xlsx
- tableS4footprinting.xlsx
- tableS3DEG.xlsx
- figureS1QC.tif
- figureS2BINDetect0209.tif
- tableS2DA.xlsx
- tableS5CHIPseqvalidationApr16.xlsx
- SupplementaryInformation.docx

## Data Availability

Single cell RNA-seq, single nucleus ATAC-seq, and ChIP-seq data have been deposited at GEO and will be publicly available under series accession number GSE283905, GSE283906, and GSE289039 upon publication. This study did not generate new unique reagents. Further information and requests should be directed to and will be fulfilled by the lead contacts, Iliya Lefterov, MD., Ph.D. (iliyal@pitt.edu) or Radosveta Koldamova, MD., Ph.D. (radak@pitt.edu).

## Abbreviations

NR: Nuclear Receptors
TF: Transcription factors
RXR: Retinoid X Receptors
LXR: Liver X Receptors
scRNA-seq: single cell RNA sequencing
snATAC-seq: single nucleus ATAC sequencing
Bexa: Bexarotene
Veh: Vehicle
APP/PS1: APP/PS1ΔE9 [B6.Cg-Tg(APPswe,PSEN1dE9)85Dbo/Mmjax]
DPBS: Dulbecco’s phosphate buffered saline
BSA: Bovine serum albumin
UMIs: Unique Molecular identifiers
P.C.: Principal component
TSS: Transcription start site
L.R.: Logistic regression
FC: Fold change
VSMC: Vascular smooth muscle cells
CREs: Cis-regulatory elements
DEGs: Differentially expressed genes
GO: Gene ontology
Sox: SRY-box transcription factors
DAM: Disease-associated microglia
PD: Parkinson’s disease
HD: Huntington’s disease
PrD: Prion disease
ALS: Amyotrophic Lateral Sclerosis
